# The relationship between physical exercise and suicidal ideation in college students: chain mediating effect of basic psychological needs satisfaction and sense of meaning in life

**DOI:** 10.3389/fpsyg.2025.1450031

**Published:** 2025-06-26

**Authors:** Yayi Ou, Kelei Guo, Yueming Cheng

**Affiliations:** School of Physical Education and Health, Zhaoqing University, Zhaoqing, China

**Keywords:** physical exercise, basic psychological need satisfaction, sense of meaning in life, suicidal ideation, college students

## Abstract

**Objective:**

To explore the relationship between physical exercise and suicidal ideation of college students, and the mediating role of basic psychological needs satisfaction and sense of meaning in life.

**Methods:**

A stratified cluster random sampling method was adopted to survey 1,008 college students of Zhaoqing University from January 17th to 27th, 2024. The Physical Exercise Scale, Suicidal Ideation Scale, Basic Psychological Needs Satisfaction Scale, and Life Meaning Scale were used to assess physical exercise, suicidal ideation, basic psychological needs satisfaction, and sense of meaning in life, respectively. Data were statistically analyzed using descriptive statistics, correlation analysis, regression analysis, and mediation analysis using SPSS (IBM Corp.).

**Results:**

Physical exercise was negatively associated with suicidal ideation (*r* = −0.26, *p* < 0.01). A significant direct effect was observed between physical exercise and suicidal ideation (*β* = −0.17, *p* < 0.01, 95% CI [−0.24, −0.11]). Physical exercise positively predicted both basic psychological needs satisfaction (*β* = 0.27, *p* < 0.01, 95% CI [0.20, 0.32]) and sense of life meaning (*β* = 0.17, *p* < 0.01, 95% CI [0.11, 0.22]). Both basic psychological needs satisfaction (*β* = −0.17, *p* < 0.01, 95% CI [−0.24, −0.10]) and sense of meaning in life (*β* = −0.12, *p* < 0.01, 95% CI [−0.20, −0.05]) were negatively associated with suicidal ideation. Additionally, basic psychological needs satisfaction was positively associated with sense of meaning in life (*β* = 0.53, *p* < 0.01, 95% CI [0.48, 0.58]). Mediation analysis revealed that both basic psychological needs satisfaction and sense of meaning in life significantly mediated the relationship between physical exercise and suicidal ideation. The mediating effect consists of three paths: the independent mediation effect of basic psychological needs satisfaction (effect value is −0.04), the independent mediation effect of sense of meaning in life (effect value is −0.02), and the chain mediation effect of basic psychological needs satisfaction and sense of meaning in life (effect value is −0.02).

**Conclusion:**

This study found that physical exercise is negatively associated with suicidal ideation among college students. Furthermore, this relationship is explained through two key mechanisms: the mediating role of basic psychological needs satisfaction, and the sequential mediation pathway involving both basic psychological needs satisfaction and sense of meaning in life. These findings highlight the importance of promoting physical exercise to enhance mental health and mitigate suicidal ideation risk in college populations.

## 1 Introduction

The rapid development of artificial intelligence, digitalization and automation technologies is profoundly reshaping the global job market. According to the World Economic Forum’s “Jobs Report 2025” (World Economic Forum, 2020), it is estimated that by 2025, 85 million jobs will be replaced while 97 million new jobs will be created. This drastic change in employment poses a severe challenge to the mental health of college students. Recent data shows that 15.9% of college students suffer from suicidal ideation (Che and Jia, 2023), highlighting the urgency of strengthening mental health intervention for college students. In response to growing concerns about student mental health, China’s Ministry of Education and 16 other ministries issued the Opinions on Strengthening Student Mental Health Work (2023–2025) (Ministry of Education of the People’s Republic of China, 2023). The policy emphasizes improving mental health literacy, strengthening education systems, and establishing effective crisis prevention and intervention mechanisms. University students, often lacking emotional regulation skills, are particularly vulnerable to anxiety and depression when facing stress, increasing the risk of suicidal ideation—a key predictor of suicidal behavior (Ma M. et al., 2022; Ma Z. et al., 2022). Although existing studies have shown that physical exercise is beneficial to improving mental health, most of them have focused on a single dimension and have less exploration of the underlying psychological mechanisms. In particular, the role of fulfilling basic psychological needs remains insufficiently studied. The self-determination theory points out that individuals are innatively endowed with three basic psychological needs: autonomy, competence and relationality, and these needs play a key role in psychological adaptation and well-being (Deci and Ryan, 2000). Studies show that physical activities help meet these needs, thereby alleviating negative emotions and enhancing psychological resilience (Chen et al., 2021a; Chen et al., 2021b; Li and Zhang, 2022). The theory of meaning construction further holds that the sense of meaning in life is an important protective factor against psychological disorders and suicidal ideation. Compared with the traditional single-factor mental health model, the two-factor model takes into account both positive and negative psychological indicators simultaneously, providing a more comprehensive research perspective. Given that university students represent a core talent pool for future societal development, this study integrates Self-determination theory, Meaning-making theory, and the Two-factor model of mental health to systematically examine how physical exercise influences suicidal ideation among university students. This study aims to explore the mediating role of basic psychological needs satisfaction and sense of meaning in life, and to provide theoretical insights for the research and policy direction supporting mental health in higher education.

### 1.1 Physical exercise and suicidal ideation

Physical exercise refers to planned physical activity involving exercise load, conducted for fitness, recreation, healthcare, rehabilitation, and psychological benefits, with the primary goals of enhancing physical fitness, improving mental health, and maintaining bodily functions (Xi, 2004). Xie (2004) identified physical exercise as an effective strategy for alleviating anxiety and other negative emotions. Compared with the same age, athletes show more courage, will, and emotional stability. Relevant studies show that physical exercise can not only improve mood and improve the quality of life, but also long-term sports training can lead to a psychological precocious phenomenon (Schuch et al., 2016). Numerous studies have explored the relationship between physical exercise and suicidal ideation or behavior. Sports psychology research demonstrates that physical exercise confers psychological benefits including enhanced self-efficacy, reduced stress reactivity, and improved emotional regulation, which may collectively mitigate suicidal ideation (Gao and Ren, 2019). In their study of 4,728 American college students, Brown and Blanton (2002) observed an inverse relationship between low-intensity physical exercise and suicidal behavior among male participants. In addition, in the investigation of physical exercise and suicidal ideation among 10,530 students, Brown et al. (2007) found that there was a negative correlation between physical exercise and suicidal ideation. There is strong evidence that physical activity is an under-studied, no-side-effect way to prevent suicide, and that physical activity can act as a depressant in several important suicide risk factors, including depression (Schuch et al., 2016), anxiety (Stubbs et al., 2017), and psychiatric disorders (Firth et al., 2015; Rosenbaum et al., 2014). Studies have shown that individuals who regularly participate in physical exercise show higher enthusiasm for life and hope for the future (Zhang et al., 2021). At the same time, the benefits of physical exercise will also inspire people to face the unknown life with a positive attitude (Liu et al., 2021). Previous research on football summer camp participants revealed a significant negative correlation between physical exercise and suicidal ideation (Gao and Ren, 2019). A Korean study of 872 high school students across eight regions similarly supported these findings (Lee and Ji, 2018). Therefore, we propose hypothesis 1: Physical exercise is negatively correlated with suicidal ideation.

### 1.2 Mediating role of basic psychological needs satisfaction

Basic psychological needs refer to the inner psychological needs that are closely related to one’s own growth, including relational needs, autonomous needs and competency needs (Wu et al., 2022). Self-determination theory is proposed in the field of psychology, which emphasizes the degree of self-determination of human behavior and divides motivation into non-motivation, external motivation, and internal motivation according to the degree of self-determination (Deci and Ryan, 2000). Basic psychological needs theory is an important part of self-determination theory. The three dimensions of competency need (ability need), relationship need (association need), and autonomy need are the most indispensable for the healthy growth of human beings (Yu, 2020). Relevant studies have pointed out that the satisfaction of basic psychological needs is crucial to the healthy development of individuals. When individuals’ basic psychological needs are satisfied, they are more likely to demonstrate positive behaviors and attitudes while exhibiting greater resilience in facing challenges (Gillison et al., 2019). However, when these fundamental needs remain unfulfilled, individuals tend to develop negative emotions or problematic behaviors (Feng and Zhang, 2021). Importantly, physical exercise has been shown to enhance the satisfaction of basic psychological needs, thereby contributing to the maintenance of optimal psychological states (Guo et al., 2023). Zhang D. D. (2011), Zhang G. (2011), and Liang (2018) examined the motivation behind physical exercise, highlighting that individuals engage in and sustain exercise behaviors to fulfill psychological needs and generate motivation. Specifically, their research demonstrates that physical exercise facilitates the satisfaction of basic psychological needs, thereby serving as a key driver of participation and adherence.

Relevant studies have confirmed that the satisfaction of basic psychological needs is closely related to suicidal ideation. When the satisfaction of basic psychological needs is in an ideal state, it will stimulate individual potential and motivation, and make individual physical and mental health develop in a beneficial direction, such as reducing depression and apathetic behavior (Ferrand et al., 2015). Extensive research has demonstrated that physical exercise can enhance individuals’ positive affect, life satisfaction, and psychological resilience, thereby strengthening positive mental health. Notably, individuals with higher levels of positive mental health exhibit relatively lower incidence of suicidal ideation even when experiencing psychopathological symptoms (Teismann et al., 2017). On the contrary, when the basic psychological needs are not met for a long time, it will hinder the healthy development of individuals, and even lead to the emergence of bad behaviors such as self-injury and suicide (Wu et al., 2018). The higher the level of satisfaction of the basic psychological needs of individuals, the less suicidal ideation and suicidal behaviors will occur (Britton et al., 2014; Wu et al., 2020). Therefore, we propose hypothesis 2: Basic psychological needs satisfaction plays a mediating role between physical exercise and college students’ suicidal ideation.

### 1.3 Mediating role of sense of meaning in life

Sense of meaning in life refers to an individual’s feeling that his or her life is understood, guided by a worthy purpose, and that life is worthwhile (George and Park, 2016). Meaning Construction Theory emphasizes individuals’ creation of personal significance through environmental interactions. Research indicates that engaging in positive behaviors such as physical exercise enables individuals to cognitively reframe life experiences, through which they enhance their sense of meaning in life. Specifically, sports participation provides rich experiential engagement and reflective opportunities that facilitate self-discovery of personal values and life purposes, ultimately elevating existential significance (Liu, 2024). Relevant studies have pointed out that sports have a significant impact on the sense of meaning of life, and Chinese scholars generally believe that college students’ physical exercise has a predictive effect on the sense of meaning of life (Xia et al., 2023). Liu et al. (2023) pointed out that there is a significant positive correlation between college students’ physical exercise, mental toughness, exercise self-efficacy, and sense of meaning in life. Physical exercise can directly predict college students’ sense of meaning in life, and there is a highly significant positive correlation between college students’ physical exercise and sense of meaning in life. Takkinen et al. (2001) found in their longitudinal study that physical exercise positively influenced both sense of meaning in life and self-rated health/function, while a stronger sense of meaning reciprocally promoted exercise engagement. Research of Marconcin (2016) demonstrated that physical exercise significantly influenced students’ self-efficacy, meaning in life perceptions, activity levels, sedentary behavior, and balance capabilities. As one of the important indicators of mental health, the sense of meaning of life is closely related to individual life satisfaction. The positive sense of life meaning can promote the change of individual lifestyle and make individuals enjoy life with a more optimistic attitude. At the same time, individuals with a high sense of life meaning will have a high evaluation of their own abilities and values, and thus experience higher life satisfaction (Gao et al., 2021). Therefore, we propose hypothesis 3: Sense of meaning in life plays a mediating role between physical exercise and college students’ suicidal ideation.

### 1.4 Chain mediating role of basic psychological needs satisfaction and sense of meaning in life

Relevant research suggests that the sense of meaning in life is a process in which an individual tends to be complete, and the level of the sense of meaning of life is the key to whether an individual can obtain happiness (Gagné, 2003). Only when an individual’s basic psychological needs are satisfied and his sense of meaning of life is realized can he experience a high level of happiness. In the basic psychological needs meeting model, only after the individual interacts with the environment and obtains the basic psychological needs meeting, will the individual get a sense of meaning in life. If the basic psychological needs cannot be met, the individual will feel intense meaninglessness and pain (Chang and Lee, 2018). Ryan and Deci (2000) pointed out that basic psychological needs are correlated with a sense of life meaning, and basic psychological needs can positively predict their sense of life meaning. The chain mediating effect between the satisfaction of basic psychological needs and the sense of meaning in life is an important way for physical exercise to reduce suicidal ideation among college students. Specifically, physical exercise can indirectly affect suicidal ideation through the mediation of basic psychological needs satisfaction and sense of meaning in life. Previous studies have shown that there is a positive correlation between basic psychological needs satisfaction and sense of meaning in life. Research has demonstrated that physical exercise indirectly enhances individuals’ sense of meaning in life by satisfying fundamental psychological needs, including autonomy, competence, and relatedness (Song, 2023). Liu et al. (2013) proposed that external environmental stimuli influence individuals’ basic psychological needs, which in turn affect self-determined motivation and ultimately shape one’s sense of meaning in life. Given that university students represent a core talent pool for future societal development, this study integrates Self-determination theory, Meaning-making theory, and the Two-factor model of mental health to systematically examine how physical exercise influences suicidal ideation among university students. Therefore, we propose hypothesis 4: Basic psychological needs satisfaction and sense of meaning in life play a chain mediating role between physical exercise and college students’ suicidal ideation.

## 2 Methods

### 2.1 Subjects

The research subjects were all students who had received physical training. During the test, the consent of both the teacher and the students was obtained. This research was conducted in accordance with the Declaration of Helsinki. All participants were informed of the purpose and characteristics of the study. Their participation was voluntary and they signed the informed consent form. This study employed a stratified cluster random sampling method to ensure the representativeness of the sample across academic years and departments. Specifically, the stratification is based on the academic year (from the first year to the fourth year). Five classes are randomly selected from each grade of the different departments of Zhaoqing University. The professional distribution includes 12 majors such as physical education, art, science and engineering, and liberal arts, with a total of 1,138 students. The questionnaire survey was conducted between January 17 and January 27, 2024, during classroom sessions using an online survey format. The administration was scheduled during regular class hours and monitored by course instructors to ensure procedural consistency. Participants completed the survey anonymously and voluntarily, with a response time of approximately 15 to 25 min. Questionnaires with excessively short completion times (e.g., answering each question in less than 5 s) or with incomplete responses were excluded. After eliminating 130 invalid questionnaires due to missing data, inconsistent responses, or insufficient answering time, a total of 1,008 valid questionnaires were retained, yielding an effective response rate of 88.56%.

### 2.2 Measurement

#### 2.2.1 Physical exercise

The physical exercise scale for college students revised by Wu (2016) was adopted, which was adapted from the physical exercise commitment intention scale compiled by Chen et al. (2006). The present scale consists of 8 questions, of which 4 are physical exercise commitment (for example, it is difficult for me to quit physical exercise) and 4 are physical exercise persistence (for example: I prefer to insist on physical exercise), the answers were all scored by Likert 5 points, from “strongly disagree,” “disagree,” “neither agree nor disagree,” “agree” and “strongly agree,” the score was 1–5, and the total score represented the physical exercise level of the subjects. The higher the total score, the higher the participant’s level of physical exercise (Jiang et al., 2018). In this study, the Cronbach’s *α* coefficient of the scale was 0.94. The scale has demonstrated good reliability among Chinese college students. For example, Wu (2016) validated the scale in his study, and the results indicated good internal consistency and structural validity. Additionally, Jiang et al. (2018) used the scale in their research on the relationship between physical exercise and mental health, and their empirical study supported its applicability to the college student population.

#### 2.2.2 Basic psychological needs satisfaction

The 9-item Basic Psychological Needs Satisfaction Scale developed by Sheldon and Niemiec (2006) was used to measure basic psychological needs satisfaction. The scale consists of 9 items (e.g., in my life, my choices are based on my true interests and values), including 3 dimensions of autonomy, competence, and relationality. All items are scored in a positive way, using a 7-point rating from 1 (unusual and inconsistent) to 7 (very consistent). The average score of the 3 items of each need is divided into the degree of satisfaction of the need, and the average score of the 9 items is divided into the total need satisfaction. The higher the score, the higher the degree of satisfaction. In this study, the Cronbach *α* coefficient of this scale was 0.90. Du et al. (2020) conducted a survey with 291 university students, and the results showed that an exploratory factor analysis yielded a three-factor structure, explaining a cumulative total of 64.73% of the total variance. This indicates that the scale has good reliability in the Chinese college student population.

#### 2.2.3 Sense of meaning in life

This study used the Life Meaning Scale developed by Steger et al. (2006) and revised by Wang (2013). The scale contains 10 questions, including two dimensions of sense of meaning and sense of meaning seeking, and each dimension includes 5 items (for example: I am looking for a purpose or mission in my life). The scale adopts a Likert 7-point scoring method, from 1 “completely inconsistent” to 7 “completely consistent,” and the total score indicates the subjects’ sense of life meaning. The higher the score, the greater the sense of meaning in life. This scale has been proven to be highly applicable in a large number of Chinese college students (Bai, 2019). In this study, the Cronbach *α* coefficient of this scale was 0.91.

#### 2.2.4 Suicidal ideation

The level of suicidal ideation was assessed using the Suicidal Behavior Screening Questionnaire developed by Yang et al. (2021). The questionnaire includes two items related to suicidal ideation, such as: “Do you wish to end your life passively through external forces?” Responses are rated on a 5-point Likert scale, where 1 indicates “never” and 5 indicates “almost every day in the past week.” Higher scores indicate stronger suicidal ideation. Although this brief tool has good acceptability and ease of use in practical applications, its reliability and validity have been verified in the Chinese university student population. Related research points out that the tool demonstrates high internal consistency and construct validity in university student samples (Zhao et al., 2023). In this study, the Cronbach’s α coefficient of the scale was 0.80, indicating good reliability.

### 2.3 Statistical analysis

The initial data were exported and analyzed using SPSS 26.0 software. After organizing the data, internal consistency reliability analysis was conducted using Cronbach’s alpha to assess reliability. Harman’s single-factor method was used to test the standard deviation of the data. The descriptive statistics on the scores of each scale and the Pearson correlation analysis were conducted using SPSS 26.0 software. Model 6 in the PROCESS plug-in and Bootstrap method by repeated sampling 5,000 times was used to test the mediating role of basic psychological needs satisfaction and sense of meaning in life between physical exercise and suicidal ideation. Variables are expressed as mean (M) ± standard deviation (SD). In this study, the significance level was set as *p* < 0.05.

## 3 Results

### 3.1 Common method deviation test

The data in this study were collected through self-reported questionnaires. As all participants were from the same university, the results may have been affected by common method bias. To address this concern, a Harman’s single-factor test was conducted. The results revealed that five factors had eigenvalues greater than 1, with the first factor accounting for 34.56% of the total variance, which is below the critical threshold of 40%.

### 3.2 Demographic characteristics

Among the valid respondents, 373 were male and 635 were female. In terms of academic level, the sample included 267 freshmen, 171 sophomores, 161 juniors, and 409 seniors. Participants ranged in age from 18 to 22 years, with a mean age of 20.36 years (SD = 1.33).

### 3.3 Descriptive statistics and correlation analysis

**Figure 1:**
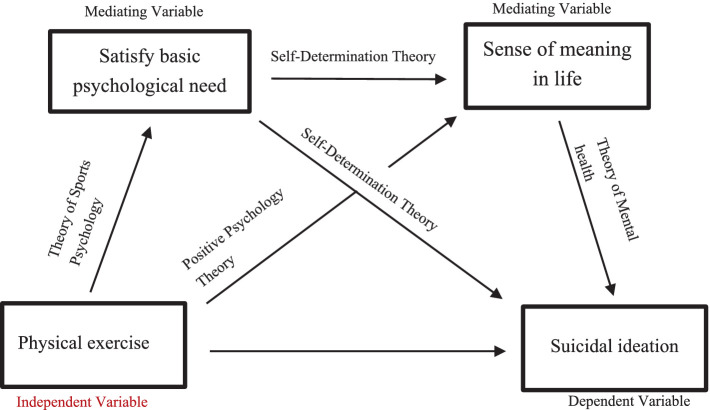
Theoretical framework of the chain mediation model.

Table 1 shows that physical exercise is positively correlated with basic psychological needs satisfaction, sense of meaning in life, and negatively correlated with suicidal ideation. Basic psychological needs satisfaction positively correlated with a sense of meaning in life and negatively correlated with suicidal ideation (Figure 1).

**Table 1:** Means, standard deviations, and correlations among variables.

Variable	*M*	SD	1	2	3	4
1. Physical exercise	27.61	6.12	1			
2. Basic psychological needs satisfaction	43.36	4.35	0.27**	1		
3. Sense of meaning in life	47.42	5.40	0.30**	0.59**	1	
4. Suicidal ideation	2.52	1.17	−0.26**	−0.26**	−0.25**	1

*N* = 1,008. ** *p* < 0.01.

### 3.4 Mediation effect test

**Figure 2:**
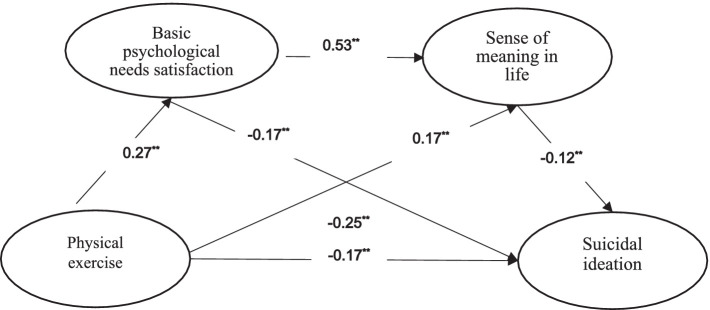
Chain mediation model of basic psychological needs satisfaction and sense of meaning in life between physical exercise and suicidal ideation (**p < 0.01).

The mediating effect was examined with basic psychological needs satisfaction and sense of meaning in life as the mediating variables, physical exercise as the independent variable, and suicidal ideation as the dependent variable. The PROCESS (Version 4.0) macro model 6 (Hayes, 2018) was adopted to conduct the mediation effect test, and a non-parametric percentile Bootstrap test (repeated sampling 5,000 times) with a 95% confidence interval (CI) was selected. The results indicated that physical exercise was negatively associated with suicidal ideation, *β* = −0.17, *p* < 0.01, CI [−0.24, −0.11], supporting Hypothesis 1. Physical exercise was positively associated with basic psychological needs satisfaction, *β* = 0.27, *p* < 0.01, CI [0.20, 0.32]; basic psychological needs satisfaction was negatively correlated with suicidal ideation, *β* = −0.17, *p* < 0.01, CI [−0.24, −0.10], supporting Hypothesis 2. Physical exercise was positively correlated with sense of meaning in life, *β* = 0.17, *p* < 0.01, CI [0.11, 0.22]; sense of meaning in life was negatively associated with suicidal ideation, *β* = −0.12, *p* < 0.01, CI [−0.20, −0.05], supporting Hypothesis 3. Furthermore, basic psychological needs satisfaction was positively associated with sense of meaning in life, *β* = 0.53, *p* < 0.01, CI [0.48, 0.58], supporting hypothesis 4. The mediating effect test (see Table 2; Figure 2) shows that the chain mediating effect of basic psychological needs satisfaction and sense of meaning in life between physical exercise and suicidal ideation is significant.

**Table 2:** Mediating effect test and effect size.

Path	Effect	The proportion of mediation in the total effect	95% CI	
			*LL*	*UL*
Physical exercise → Basic psychological needs satisfaction→ Suicidal ideation	−0.04	−0.04/−0.08 = 50%	−0.08	−0.02
Physical exercise → Sense of meaning in life → Suicidal ideation	−0.02	−0.02/−0.08 = 25%	−0.04	−0.01
Physical exercise → Basic psychological needs satisfaction → Sense of meaning in life → Suicidal ideation	−0.02	−0.02/−0.08 = 25%	−0.03	−0.01
Total effect	−0.08		−0.12	−0.05

CI, confidence interval; LL, lower limit; UL, upper limit.

The values presented in Figure 2 are the values of *β*, which are obtained using the stepwise regression method. *β* is the standardized coefficient.

## 4 Discussion

This study explores the relationship between physical exercise and college students’ suicidal ideation, as well as the mediating effects of basic psychological needs satisfaction and sense of meaning in life. The results show that physical exercise can not only positively affect suicidal ideation, but also indirectly affect suicidal ideation through the independent mediating effect of basic psychological needs satisfaction and sense of meaning in life. Furthermore, physical exercise also affect suicidal ideation through the chain mediation of basic psychological needs satisfaction and sense of meaning in life. In addition, this study further explains the causes of the effect of physical exercise on suicidal ideation and has certain enlightenment signiffcance for the prevention and intervention of suicidal ideation among college students.

### 4.1 Physical exercise and suicidal ideation

This study found that physical exercise has a significant negative correlation with college students’ suicidal ideation, consistent with previous research (Gao and Ren, 2019; Brown et al., 2007). From a physiological perspective, physical exercise promotes the secretion of dopamine (Monteiro-Junior et al., 2015), which may reduce suicidal ideation (Roy et al., 1992). Additionally, physical exercise increases heart rate variability (Peng, 2013), which has been linked to lower levels of suicidal ideation (Diekstra and Garnefski, 1995). Moreover, physical exercise has been shown to improve mental health, reduce psychological tension, and increase psychological resilience, which helps lower the risk of suicidal ideation (Fan et al., 2005; Sun and Yang, 2015). Individuals with better mental health have lower levels of suicidal ideation (Li et al., 2019). Physical exercise may influence suicidal ideation both directly and indirectly, potentially mediated by factors such as general self-efficacy (Chen et al., 2021a; Chen et al., 2021b). Given these findings, educational institutions should prioritize the development of physical education curricula by increasing class hours and diversifying sports activities, actively encouraging student participation, and raising awareness of the importance of physical exercise. While these initiatives can enhance mental health and reduce suicidal ideation, further empirical studies are needed to establish optimal exercise parameters (duration, intensity, and frequency).

### 4.2 Independent mediating effects of basic psychological need satisfaction and sense of meaning in life

In addition to the direct impact of physical exercise on suicidal ideation, this study identified that both basic psychological need satisfaction and sense of meaning in life play independent mediating roles. The satisfaction of basic psychological needs, such as autonomy, competence, and relatedness, was positively correlated with physical exercise (Zhang D. D., 2011; Zhang G., 2011; Liang, 2018) and negatively correlated with suicidal ideation (Wu et al., 2022; Britton et al., 2014). As self-determination theory (SDT) suggests, the fulfillment of these basic psychological needs through physical activity fosters psychological well-being and reduces emotional stress and anxiety, which are risk factors for suicidal ideation (Deci and Ryan, 2000). Similarly, a sense of life meaning, which is positively linked to physical exercise (Liu et al., 2023; Wu et al., 2009), plays a mediating role in reducing suicidal ideation. Physical exercise triggers changes in neurotransmitter levels, such as endorphins and catecholamines, leading to enhanced emotional states and a stronger sense of life meaning (Ding and Fan, 2002; Ding et al., 2016). These improved emotional states promote psychological resilience, emotional regulation, and a more positive outlook on life, reducing suicidal ideation (Zeng et al., 2018).

### 4.3 Chain mediation of basic psychological needs satisfaction and sense of meaning in life

The most significant finding in this study is the identification of a chain mediation effect, where basic psychological needs satisfaction and sense of meaning in life mediate the relationship between physical exercise and suicidal ideation. Specifically, physical exercise indirectly influences suicidal ideation through the sequential mediation of basic psychological need satisfaction and a sense of life meaning. This chain mediation pathway aligns with both Maslow’s hierarchy of needs theory and Self-determination theory, which highlight that meeting fundamental psychological needs enhances psychological health and fosters a sense of purpose and meaning in life (Deci and Ryan, 2000). The empirical results confirm that physical exercise fulfills individuals’ core psychological needs (autonomy, competence, and relatedness), which in turn cultivates a sense of life meaning. This positive experience serves as a psychological buffer against suicidal ideation. Furthermore, this study provides valuable insight into how the fulfillment of psychological needs through physical exercise enhances life meaning, thereby reducing the risk of suicidal ideation. Compared to single mediation models, the chain mediation model offers a more comprehensive explanation of the psychological mechanisms at play, reflecting a “motivation-to-meaning” process. This chain mediation pathway has important implications for suicide prevention. By fostering physical activity, educational institutions can simultaneously meet students’ basic psychological needs satisfaction and enhance their sense of meaning in life, providing a dual-layered psychological defense against suicidal ideation. These findings deepen our understanding of the interplay between physical exercise, psychological needs, and mental health, offering a robust theoretical framework for intervention strategies.

### 4.4 Limitations and prospects

First, the study relies on self-report data, which may introduce biases such as social desirability bias or recall bias. Future research could incorporate a combination of self-reports and external evaluations to enhance the reliability and validity of the data. Meanwhile, to further strengthen the psychometric foundation of the measurement tool, future studies may consider using a more comprehensive and well-structured suicide ideation scale (such as the Beck Scale for Suicide Ideation, BSI) to enhance the ability to capture and interpret the characteristics of suicidal thoughts in university students. Second, the study uses a cross-sectional design, which limits the ability to draw causal conclusions between physical exercise and suicidal ideation. Future studies could employ longitudinal tracking or experimental intervention designs to better understand the causal relationships and mechanisms involved. Third, this study only considers the mediating roles of basic psychological needs satisfaction and sense of meaning in life, but does not explore other potential mediating variables. Factors such as physical health issues, exercise adherence, and varying amounts of physical exercise may play important roles in influencing the relationship between physical exercise and suicidal ideation. Future research should explore these additional variables for a more comprehensive understanding. Additionally, the results of this study may be limited in terms of generalizability due to potential regional differences. The findings may not be applicable to other populations with different cultural, geographic, or demographic characteristics. Thus, further research in diverse contexts is needed to validate and extend these results.

## 5 Conclusion and recommendations

This study reveals that physical exercise negatively predicts suicidal ideation among college students, suggesting that active participation in physical exercise helps reduce suicidal ideation. Moreover, both basic psychological needs satisfaction and sense of meaning in life serve as key mediators in this relationship. Specifically, physical exercise not only indirectly reduces suicidal ideation by enhancing students’ fulfillment of autonomy, competence, and relatedness but also promotes a stronger sense of meaning in life, thereby supporting overall mental health. Importantly, the sequential mediation effect of basic psychological needs satisfaction followed by sense of meaning in life further illustrates that enhanced life meaning, rooted in the fulfillment of intrinsic needs, is a critical pathway through which physical exercise supports psychological well-being. These findings offer both theoretical insights and practical implications for promoting mental health in higher education. Universities should scientifically design physical education curricula by offering diverse, interest-based modules and emphasizing experiential participation. It is also important to strengthen the physical exercise infrastructure to ensure accessibility. Furthermore, incorporating “exercise intervention” programs into mental health services, such as providing exercise-based counseling or stress reduction workshops, can help students regulate their emotions and enhance their adaptability. Awareness campaigns on the psychological benefits of physical exercise should also be promoted to encourage students to view exercise as an essential tool for emotional regulation and mental health maintenance.

## Data Availability

The original contributions presented in the study are included in the article/supplementary material, further inquiries can be directed to the corresponding authors.
