# Correction: The relationship between physical exercise and suicidal ideation in college students: chain mediating effect of basic psychological needs satisfaction and sense of meaning in life

**DOI:** 10.3389/fpsyg.2025.1672053

**Published:** 2025-09-25

**Authors:** Yayi Ou, Kelei Guo, Yueming Cheng

**Affiliations:** School of Physical Education and Health, Zhaoqing University, Zhaoqing, China

**Keywords:** physical exercise, basic psychological need satisfaction, sense of meaning in life, suicidal ideation, college students

Author Yayi Ou was erroneously assigned as corresponding author. The correct corresponding author is Yueming Cheng.

The original version of this article has been updated.

